# Inhibitory Effect of Alpha-Mangostin to Dengue Virus Replication and Cytokines Expression in Human Peripheral Blood Mononuclear Cells

**DOI:** 10.1007/s13659-019-00218-z

**Published:** 2019-09-19

**Authors:** Zaenal Sugiyanto, Benediktus Yohan, Soeharyo Hadisaputro, Edi Dharmana, Catharina Suharti, Winarto, Kis Djamiatun, Fifin L. Rahmi, R. Tedjo Sasmono

**Affiliations:** 1 Faculty of Health Science, Dian Nuswantoro University, Jl. Imam Bonjol 270, Semarang, 50131 Indonesia; 2 Doctoral Program in Medical and Health Sciences, Faculty of Medicine, Diponegoro University, Jl. Prof. Sudarto SH, Tembalang, Semarang, 50275 Indonesia; 3 Eijkman Institute for Molecular Biology, Ministry of Research, Technology and Higher Education, Jl. Diponegoro 69, Jakarta, 10430 Indonesia; 4 Faculty of Medicine, Diponegoro University, Jl. Prof. Sudarto SH, Tembalang, Semarang, 50275 Indonesia

**Keywords:** Dengue, Antiviral, Mangostin, Cytokine, PBMC

## Abstract

**Abstract:**

Massive pro-inflammatory cytokines production has been correlated with the pathogenesis of severe dengue disease. The active compound of mangosteen fruit pericarps, α-mangostin, has been commonly used as traditional medicine and dietary supplement. We examined the effect of α-mangostin against dengue virus (DENV) infection in human peripheral blood mononuclear cells (PBMC) by the measurement of virus titer and TNF-α and IFN-γ cytokines concentration post infection. Increasing concentration of α-mangostin inhibited virus replication and reduced inflammatory cytokines expression at 24- and 48-h post infection. Our results support the potential use of α-mangostin as anti-antiviral and anti-inflammatory therapies in the treatment of dengue.

**Graphic Abstract:**

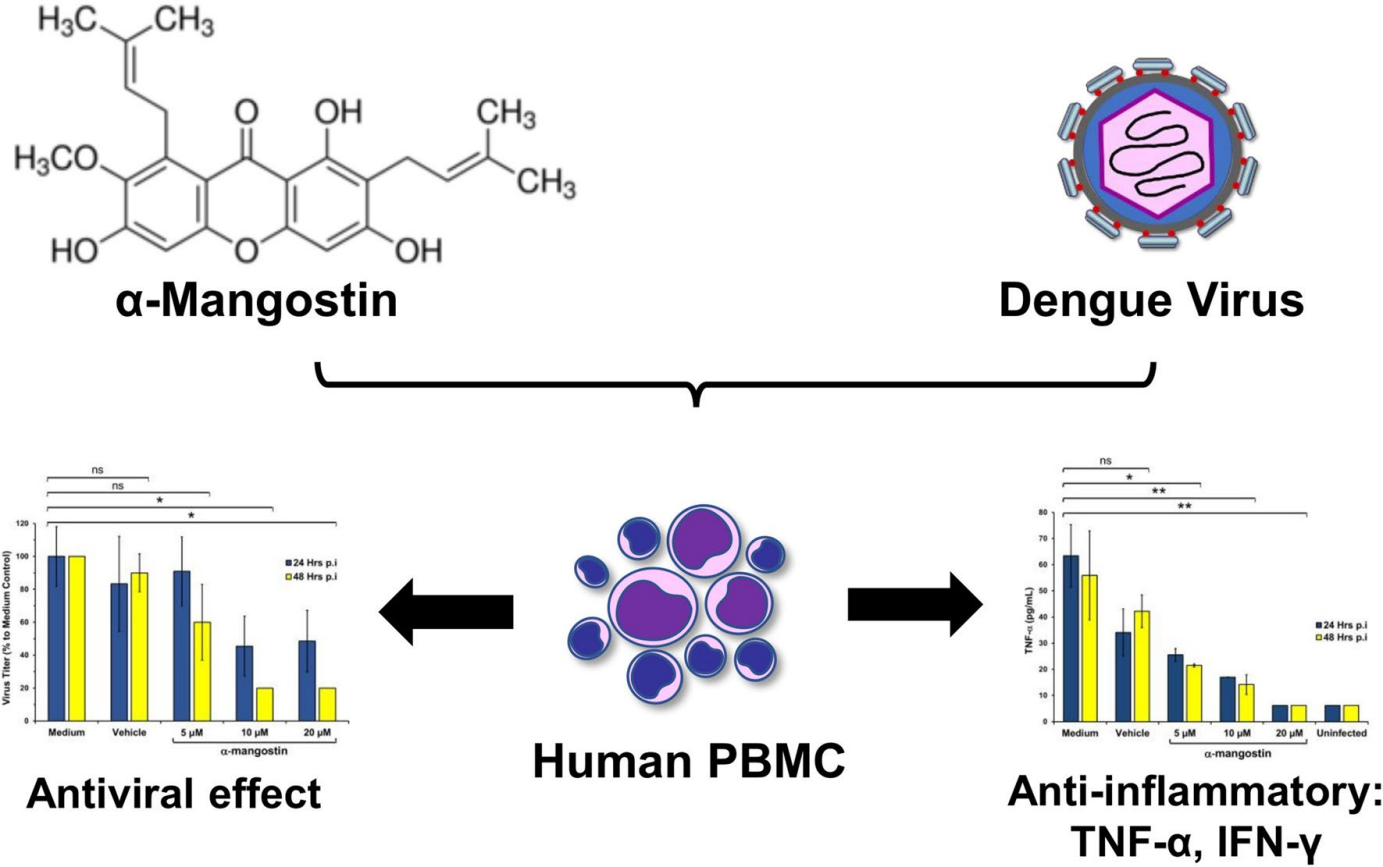

## Introduction

Dengue disease has been regarded as the most prevalent and rapidly spreading mosquito-borne viral disease of human [1]. Dengue pose a significant burden to global health with more than half of the world population living in areas with risk of dengue transmission [2]. Dengue virus (DENV), the etiological agent of dengue disease, is a member of Flaviviridae family with four serotypes (DENV-1, DENV-2, DENV-3, and DENV-4) circulating and transmitted by *Aedes* mosquitoes [3].

Infection of DENV to a susceptible human host can present as a wide range of clinical phenotypes ranging from mild manifestation (dengue fever, DF) to the more severe spectrum (dengue hemorrhagic fever, DHF and dengue shock syndrome, DSS) being defined as a syndrome of capillary leak, coagulopathy, and organ impairment and in part thought to be immune-mediated [4]. The exact mechanism of dengue pathogenesis leading to severe clinical manifestation is not fully understood, however, several risk factors may explain the progression to DHF/DSS. The antibody dependent enhancement (ADE) in secondary infection with heterotypic DENV enhance viral entry leading to increased viral load and host’s immune activation [5, 6]. In addition, secondary infection may trigger activation of serotype-cross-reactive memory T-cells, resulting in massive pro-inflammatory cytokine production leading to the so called “cytokine storm” [7]. The role of cytokines and chemokines in the pathogenesis of severe dengue has been reported [8, 9]. Using an in vitro cell infection model, the cytokine/chemokine expression profiles of DENV-infected cells has been described [10].

The past decades have witnessed the significant increase in dengue research, covering topics on dengue virology, pathogenesis, and immunology and progress in development of antivirals, vaccines, and new vector-control strategies which are important for dengue control and prevention [1]. Despite the fact, currently there are no antiviral therapies available, and the only licensed vaccine has limited use in the target population below nine years old [11]. Mangosteen (*Garcinia mangostana* Linn) is a tropical fruit that grows in Asian region, including Indonesia. The pericarps of this fruit have been traditionally used for the treatment of sicknesses such as trauma, skin infection, abdominal pain, dysentery, and wounds [12]. The mangosteen peel contains considerable amounts of biologically active compounds, such as xanthones, terpenes, anthocyanins, tannins, and phenols [13]. Many medicinal benefits are contributed by those compounds which particularly effective against oxidative damage and inflammatory response, as well as inhibition of cancer and bacterial growth [14]. Alpha mangostin (α-mangostin), one of the major xanthone compound of mangosteen, has been shown to inhibit both DENV production in vitro in hepatocellular carcinoma HepG2 and Huh-7 cell lines, and cytokine/chemokine expression in HepG2 cells [15].

Peripheral blood mononuclear cells (PBMC) has been reported to be susceptible to DENV infection and display a vigorous cytokine/chemokine response upon viral infection [16–18]. Variable profiles of cytokines/chemokines gene expression were also observed in patients during phases of dengue illness [9]. In this study, we characterized the antiviral activity of α-mangostin and the expression profiles of two cytokines involved in the inflammatory immune response to DENV infection, i.e., tumor necrosis factor alpha (TNF-α) and interferon gamma (IFN-γ). Instead of using cell lines, we used PBMCs in this study to better reflect DENV infection and the immune response against DENV infection in human.

## Results and Discussion

**Fig. 1:**
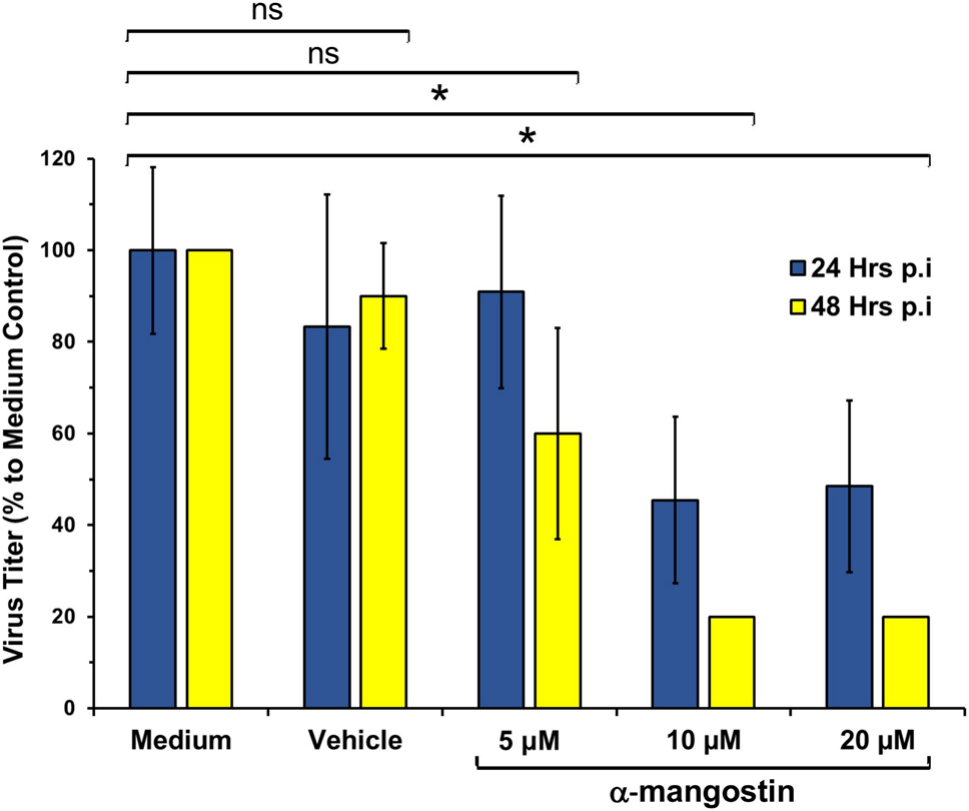
The effect of α-mangostin treatments to DENV replication in infected PBMC. Virus titer was measured using plaque assay method. Data were presented as percentage of virus titer to medium only control. Statistical significance: ns = not significant; *p < 0.05

Upon treatment with α-mangostin, the titer of viable virus in the supernatant of DENV-infected PBMC was quantified using plaque assay method to infer the quantity of propagated virus secreted to the supernatant. Compared to the medium only control, treatment of α-mangostin reduced the number of viable DENV-2 in the system (Fig. 1). A viral reduction of more than 50% was observed after treatment using 10 and 20 μM of α-mangostin at 24- and 48-h post infection, while greater reduction effect was observed at 48 h post infection. The calculated IC_50_ were 5.47 and 5.77 μM for 24- and 48-h treatments, respectively. A non-significant viral reduction was observed when vehicle control was applied. The antiviral activity of α-mangostin to DENV is in accordance with the in vitro results from Tarasuk, et al. [15].

**Fig. 2:**
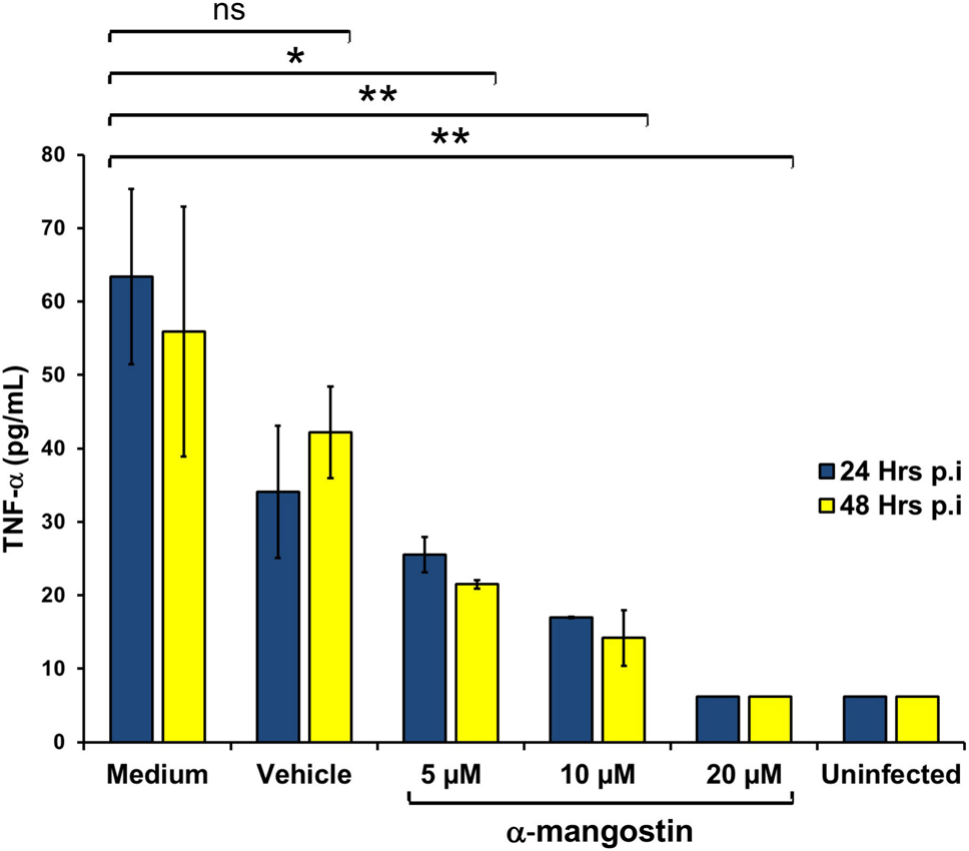
The effect of α-mangostin treatment to TNF-α expression in DENV-infected PBMC at 24- and 48-h post infection. Statistical significance: ns = not significant; *p < 0.05; **p < 0.01

**Fig. 3:**
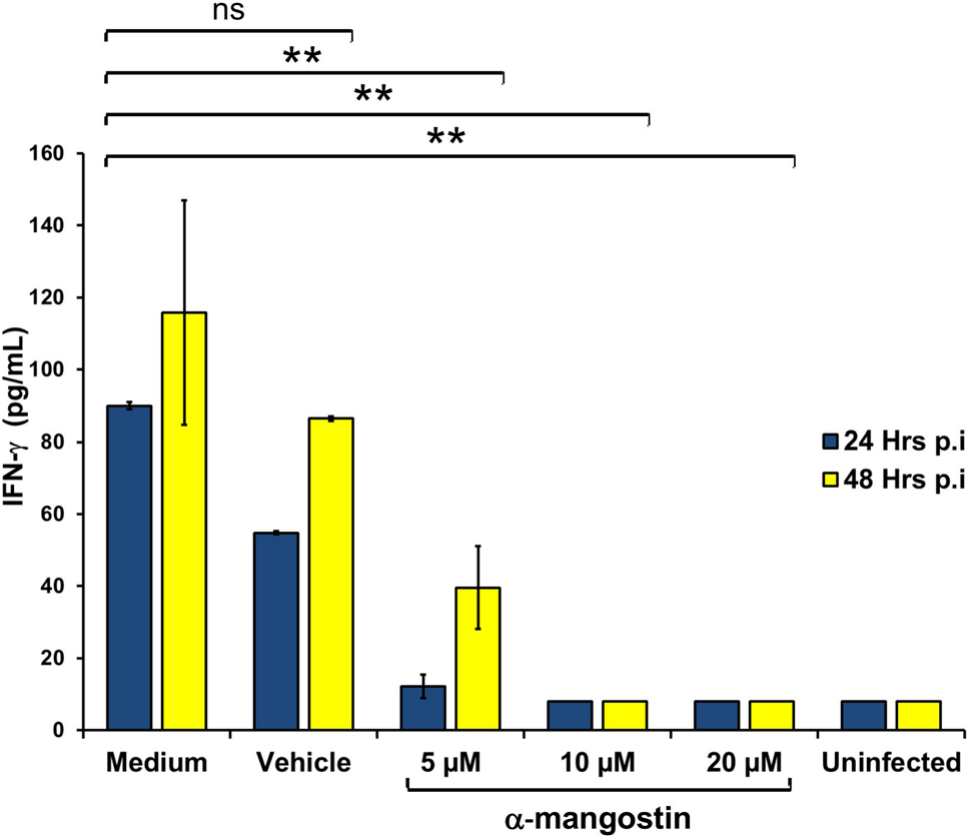
The effect of α-mangostin treatment to IFN-γ cytokine expression in DENV-infected PBMC at 24- and 48-h post infection. Statistical significance: ns, not significant; **p < 0.01

A synergistic anti-inflammatory effect also observed when cytokine expression profiles of DENV-infected PBMC was measured for IFN-γ and TNF-α using quantitative ELISA kits. Infected PBMC (as DENV in medium only without compound treatment) showed increase in cytokine expression compared to uninfected control (Figs. 2 and 3). Treatment of α-mangostin reduced the cytokines expression of both TNF-α (Fig. 2) and IFN-γ (Fig. 3) and the reduction rate was higher along with the increasing concentration of α-mangostin. Significant reduction (linear logistic regression, *p* = 0.01) of cytokine expression was observed in all three different concentrations of compound treatment and in both 24- and 48-h post infection conditions. A reduction profile was observed in vehicle control group, although not statistically significant.

In the virological part, studies reported that DENV-2 is more likely to cause severe form of dengue clinical manifestation [19, 20] and the likeliness increased in secondary infection [21]. In this study, we utilized a DENV-2 clinical isolate from a DHF patient in Semarang [22] as challenge virus. The use of wild-type DENV strain may eliminate the potential bias in using prototype virus that has been extensively manipulated in the laboratory.

TNF-α is a pro-inflammatory cytokine known to be elevated in the critical phase of dengue illness [23]. The level of TNF-α might be associated with DENV infection and related to disease severity, in which DHF patients had significantly higher levels of TNF-α than DF patients [24]. An in vivo study in mice recorded the production of high levels of TNF-α in tissues correlated with endothelial cell apoptosis and hemorrhage, along with high viral titer and macrophage infiltration [25]. IFN-γ is a key player in driving cellular immunity and involved in numerous protective functions to heighten immune responses in infections, inducing an antiviral state [26]. Related to disease severity, the level of IFN-γ was significantly increased in patients with severe clinical manifestations when compared to mild dengue [8]. Among the host inflammatory mediators, both TNF-α and IFN-γ were significantly increased in dengue with and without warning signs, in severe dengue patients compared to healthy controls [27].

The use of α-mangostin as an antiviral drug to inhibit DENV transmission is of valuable advantage especially for endemic countries where all four DENV serotypes circulating and secondary infections leading to the more severe form of disease is unavoidable. The intervention of α-mangostin in dengue patients during the acute phase of illness may reduce disease severity in relation to the increasing viral load and activation of host’s immune response through the mechanism of interfering DENV NS5 protein activity essential for DENV replication and reducing transcriptional responses of cytokines, as reported elsewhere [15]. All in all, our results support the potential use of α-mangostin as antiviral and anti-inflammatory therapies in the treatment of dengue. Future studies are needed to confirm the findings.

## Experimental Section

### Virus Strain

The DENV-2 strain SMG-SE001 of Cosmopolitan genotype (henceforth called as DENV-2) was isolated from a patient with DHF clinical manifestation from Semarang, Indonesia [22]. Virus was maintained and propagated in Vero CCL-81 cells (ATCC).

### Blood Collection and PBMC Isolation

Human PBMC fraction was collected from whole blood of a healthy donor. Involvement of human subject in this study was approved by the Research Ethics Commission of the Faculty of Medicine, Diponegoro University. The donor was negative for dengue infection based on Dengue NS1 antigen and anti-DENV IgM/IgG antibodies detection. Approximately, 20 mL of venous blood was collected using aseptic venipuncture technique and stored in heparinized tubes (Vacuette, Greiner BioOne). The blood was further diluted using Dulbecco's phosphate-buffered saline (D-PBS) (Gibco-Thermo Fisher Scientific) in a ratio of 1:2. The diluted blood was added carefully into 15 mL centrifuge tubes containing Ficoll Histopaque-1077 PLUS (GE Healthcare) solution to generate layers of blood on the top of Ficoll in a ratio of 1:1. Tubes were centrifuged for 40 min at 400×*g* in a centrifuge chamber set to 20 °C. The buffy coat ring containing PBMC was collected and pooled, washed twice with D-PBS (three times volume of PBMC suspension), centrifuged for 10 min at 100×*g* at 20 °C, and resuspended in complete RPMI 1640 medium supplemented with 10% FBS and 1% antibiotic/antimycotic (Gibco-Thermo Fisher Scientific). The number of isolated PBMC was counted using hemocytometer (Improved Neubauer) and cells were seeded in wells of 24-well plates (Corning, USA).

### DENV Infection to PBMC and Compound Treatment

A total of 1 × 10^6^ PBMC/well was cultured in 1 mL of RPMI complete medium in 24-well plates and incubated overnight at 37 °C incubator supplemented with 5% of CO_2_ for cells adaptation and resting prior to infection. Cells were then infected with DENV-2 at a multiplicity of infection (MOI) of 1 plaque forming unit (PFU)/cell giving a theoretical of one virus particle infecting each cell. PBMC infection was allowed for 90 min at 37 °C, 5% CO_2_ and performed in duplicate for each group. Infected cells were then treated with medium containing either α-mangostin, ≥ 98% (HPLC) (Sigma) or methanol solvent, analytical grade (Merck) as vehicle control (prepared as 0.1% v/v of methanol in medium), or medium only control. The preparation of 0.1% methanol in medium was equal to the level of methanol in the preparation of 100 μM of α-mangostin in the bioassay, a concentration that was five times higher than our highest test concentration (20 μM). The uninfected PBMC served as background control. The working concentration of 5, 10, and 20 μM of α-mangostin were selected and used to study the effect of different concentrations of compound treatment, according to the previous in vitro result [15]. The plates were incubated for 24 and 48 h at 37 °C incubator, 5% CO_2_, followed by the harvest of cells and supernatant. Samples were stored at − 80 °C until use.

### Viable Virus Titration Using Plaque Assay

Virus titer was quantified as viable virus in the supernatant using plaque assay method, as described elsewhere [10, 28]. Briefly, 24-well plates were seeded with BHK21 cells in RPMI complete medium at a density of 2 × 10^5^ cells/well and incubated at 37 °C incubator, 5% CO_2_. Following two days incubation period, cell monolayers were infected with 200 μL of the tenfold serially diluted supernatant from PBMC infection assay, in duplicate, and virus allowed for adsorption for 60 min at 37 °C, 5% CO_2_. For low titer of virus, a dilution of twofold was applied. Inoculated wells were then aspirated and replenished with the addition of 0.5 mL of 1% carboxymethylcellulose (CMC, Merck) overlay. Plates were incubated for five days at 37 °C, 5% CO_2_. Following the incubation period, cells were then fixed with 3.7% formaldehyde solution for 30 min and visualized by crystal violet staining. Plaques were counted and calculated by incorporating the dilution factor and titer then expressed as PFU/mL. Viral inhibition curve was plotted as percentage of virus titer in treatment group relative to the titer of medium only control.

### Cytokine Concentration Measurement Using ELISA

The measurement of human TNF-α and IFN-γ cytokines concentrations was done using Quantikine ELISA kits (R&D Systems), according to protocol specified in each kit’s manual. The measurement was using an amount of 50 μL of supernatant for TNF-α and 100 μL of supernatant used for IFN-γ. Quantitative measurement of cytokines was done utilizing the known concentration of standards available in the kit, run together with samples and results plotted as standard curve for each analyte. The kit’s limit of detection was 6.2 pg/mL for TNF-α and 8.0 pg/mL for IFN-γ.

### Statistical Analysis

Statistical analysis was done using IBM SPSS Statistics version 25 software. The independent t-Test was used to compare means between two groups. A linear logistic regression analysis was applied to compare the effect of different concentrations of compound treatments compared to control. The *p* value of < 0.05 was considered as statistically significant. Results were presented as the mean ± standard deviations (SD).
